# Clinical and immunological features associated to the development of a sustained immune humoral response in COVID-19 patients: Results from a cohort study

**DOI:** 10.3389/fimmu.2022.943563

**Published:** 2022-08-15

**Authors:** Jiram Torres-Ruiz, Julieta Lomelín-Gascón, Ana Sofía Vargas-Castro, Jaquelin Lira-Luna, Alfredo Pérez-Fragoso, Roberto Tapia-Conyer, Miroslava Nuñez-Aguirre, Beatriz Alcalá-Carmona, Abdiel Absalón-Aguilar, José Luis Maravillas-Montero, Nancy Raquel Mejía-Domínguez, Carlos Núñez-Álvarez, Marina Rull-Gabayet, Luis Llorente, Sandra Romero-Ramírez, Victor Andrés Sosa-Hernández, Rodrigo Cervantes-Díaz, Guillermo Juárez-Vega, David Eduardo Meza-Sánchez, Luis Alberto Martínez-Juárez, Linda Morales-Juárez, Lizeth Naomi López-López, José Adrián Negrete-Trujillo, Jorge Abelardo Falcón-Lezama, Rafael Ricardo Valdez-Vázquez, Héctor Gallardo-Rincón, Diana Gómez-Martín

**Affiliations:** ^1^ Department of Immunology and Rheumatology, Instituto Nacional de Ciencias Médicas y Nutrición Salvador Zubirán, Mexico City, Mexico; ^2^ Operative Solutions, Carlos Slim Foundation, Mexico City, Mexico; ^3^ Sección de Estudios de Posgrado e Investigación, Escuela Superior de Medicina-Instituto Politécnico Nacional (IPN), Mexico City, Mexico; ^4^ Laboratorio de Inmunoquimica 1, Posgrado en Ciencias Químicobiológicas, Escuela Nacional de Ciencias Biológicas, Instituto Politécnico Nacional, Mexico City, Mexico; ^5^ Universidad Nacional Autónoma de México, Facultad de Medicina, Mexico City, Mexico; ^6^ Internal Medicine Department, Instituto Nacional de Ciencias Médicas y Nutrición Salvador Zubirán, Mexico City, Mexico; ^7^ Red de Apoyo a la Investigación, Universidad Nacional Autónoma de México e Instituto Nacional de Ciencias Médicas y Nutrición Salvador Zubirán, Mexico City, Mexico; ^8^ Clinical Research, London School of Hygiene and Tropical Medicine, London, United Kingdom; ^9^ Temporary COVID-19 Hospital, Hipódromo de las Américas, Mexico City, Mexico; ^10^ Division of Health Sciences, Juárez Autonomous University of Tabasco, Tabasco, Mexico; ^11^ Centro Universitario de Ciencias de la Salud (CUCS), Universidad de Guadalajara, Guadalajara, Mexico

**Keywords:** SARS-CoV-2, humoral response, COVID-19, lymphopenia, anti-cellular antibodies

## Abstract

**Background:**

Until now, most of the research addressing long-term humoral responses in coronavirus disease 2019 (COVID-19) had only evaluated the serum titers of anti-severe acute respiratory syndrome coronavirus 2 (SARS-CoV-2) IgGs, without the assessment of the baseline antiviral clinical and immune profile, which is the aim of this study and may be the key factor leading to a broad and sustained antibody response.

**Methods:**

We included 103 patients with COVID-19. When the patients sought medical attention (baseline), a blood sample was drawn to perform immunophenotype of lymphocytes by flow cytometry. The patients were assessed 15 days after baseline and then every month until the third month, followed by a last visit 6 months after recruitment. We evaluated the anti-SARS-COV-2 IgG at all time points, and the serum levels of cytokines, chemokines, anti-cellular (AC) antibodies and neutrophil extracellular traps were also assessed during the follow-up. The primary outcome of the study was the presence of a sustained immune humoral response, defined as an anti-SARS-CoV-2 IgG titer >4.99 arbitrary units/mL in at least two consecutive measures. We used generalized lineal models to assess the features associated with this outcome and to assess the effect of the changes in the cytokines and chemokines throughout time on the development of a sustained humoral immune response.

**Results:**

At baseline the features associated to a sustained immune humoral response were the diagnosis of critical disease, absolute number of lymphocytes, serum IP-10, IL-4, IL-2, regulatory T cells, CD8^+^ T cells, and positive AC antibodies. Critical illness and the positivity of AC antibodies were associated with a sustained humoral immune response after 3 months, whilst critical illness and serum IL-13 were the explanatory variables after 6 months.

**Conclusion:**

A sustained immune humoral response is strongly related to critical COVID-19, which is characterized by the presence of AC antibodies, quantitative abnormalities in the T cell compartment, and the serum cytokines and chemokines during acute infection and throughout time.

## Introduction

Severe acute respiratory syndrome (SARS-CoV-2) has affected 78 million individuals and is responsible for over 1.7 million deaths to date (1). After its emergence, initial scientific efforts were focused on the understanding of the acute antiviral immune response (2), but currently, the long-term cellular and humoral immune responses against SARS-CoV-2 have become relevant (3). In this regard, there is an increased interest in the detection of a sustained humoral immune response as a marker of anti-SARS-CoV-2 vaccination, as well as a key risk factor for re-infection (3, 4), and for the development of post-coronavirus disease 2019 (COVID-19) syndrome (5).

Nearly all patients with COVID-19 develop a humoral antiviral immune response (6). The magnitude of the humoral immune response is strongly correlated with the disease severity (7) and the duration of active infection (8), highlighting the importance of the initial antiviral response in the development of sustained humoral immunity. A prolonged and severe COVID-19 is the clinical consequence of a disturbed adaptive and innate antiviral immune response. In this regard, patients with COVID-19 are characterized by the expression of PD1 and CD57 in the T cell compartment, which has been related to an enhanced production of TNF, CD107a, IFN-γ, IL-2 and IL-17 (9). Many pro-inflammatory cytokines and chemokines are chemoattractant and activator of neutrophils, promoting an exuberant myeloid immune response, which is another typical feature of severe COVID-19 (10). After activation, neutrophils secrete extracellular traps (NETs), which are key drivers of COVID-19 severity, by the promotion of immunothrombosis and the cytokine storm (11). Regarding the B cell compartment, during acute SARS-CoV-2 infection, there is evidence of an extrafollicular B cell response, resulting in non-class-switched memory B cell development. Also, during acute COVID-19, some B cells enter the germinal center and differentiate into class-switched memory B lymphocytes and plasma cells (12). Although patients with severe COVID-19 have a potent inflammatory response, it is usually ineffective for viral clearance, which may lead to higher viral antigen loads with epitope spreading, resulting in a broad and robust anti-SARS-CoV-2 humoral response (12). Compared with uninfected controls, COVID-19 patients exhibit dramatic increases in autoantibody reactivity, including a high prevalence of autoantibodies against immunomodulatory proteins, such as cytokines, chemokines, complement components, and cell surface proteins, altogether leading to impaired immune function (13).

Currently, most of the research about the long-term humoral immune response in COVID-19 has been focused on the assessment of the serum titers of anti-SARS-CoV-2 IgG throughout time (14), without any insight into the dysregulated immune response at baseline, which may be the key driver of a sustained humoral immunity. In this study we aimed to explore the previously unrecognized role of the clinical and immunological profile of patients with COVID-19 during the acute infection as drivers of the development of a sustained humoral immune response. Our data contributes to the characterization of the clinical and immunological features leading to a broad and persistent antibody response in patients with COVID-19.

## Methods

An observational cohort study was conducted in 103 patients with COVID-19 diagnosis, which was confirmed by a positive polymerase chain reaction (PCR) test from a nasopharyngeal swab. The patients were seen at the Temporary COVID-19 Hospital, which served as the main reference center for the care of patients with COVID-19 in Mexico City. Patients were recruited between August 2020 and February 2021 and were followed up until August 2021. The Institutional Ethics and Research committees from the Temporary COVID-19 Hospital approved the study protocol (Ref. 3341). All participants provided written informed consent to participate in the study at enrollment. The present research study was in accordance with the ethical principles outlined in the Declaration of Helsinki.

When the patients sought medical attention, which corresponded to the baseline evaluation, clinical data and laboratory parameters were obtained. According to their disease severity, patients were classified into the following categories: mild/moderate (patients with or without pneumonia who also had fever and upper respiratory infection symptoms); severe (patients presenting respiratory failure with ≥30 breaths per minute, a resting oxygen saturation ≤93% or PaO_2_/FiO_2_ ≤300); or critical (patients in a state of shock, multiple organ failure or those requiring invasive mechanical ventilation) (15). The exclusion criteria were the diagnosis of cancer, chronic infections, autoimmune diseases, pregnancy, and puerperium.

A blood sample was drawn to isolate peripheral blood mononuclear cells (PBMCs) at baseline. Patients were followed up two weeks after the onset of symptoms, then every month until the third month, and at 6 months post recruitment. Serum samples were collected in all patients at baseline and at all visits. Sera were stored at −80 °C until analyses. Immunophenotyping of T and B cell subsets was performed by multiparametric flow cytometry, and serum samples were analyzed for detection of anti-SARS-CoV-2 IgG antibodies, anti-cellular (AC) antibodies, chemokines/cytokines, and circulating neutrophil extracellular traps (NETs), as described in detail below. The primary outcome of the study was the development of a sustained humoral immune response, which was defined as the positivity of anti-SARS-CoV-2 IgG antibodies in at least two consecutive measurements. Positivity was defined as a titer higher than 4.99 arbitrary units (AU)/mL, as previously reported (16). We decided to use this cutoff point because this titer correlated with the neutralizing activity and can be widely used in many laboratories without access to the viral neutralizing assays (16). Samples processing was carried out in a blinded manner, that is, by investigators who were unaware of the study outcome. There were no missing serum samples from any patient at any timepoint and all subjects completed follow-up.

### Immunophenotyping

PBMCs were purified by density gradient from peripheral blood using Ficoll-Paque (GE Healthcare Life Sciences, Chicago, IL, USA) and centrifugation. After isolation, cells were washed twice with phosphate-buffered saline (PBS). Viability was then assessed by Zombie Aqua staining (Biolegend, San Diego, CA, USA). Then, PBS was used for washing the cells, which were incubated with the FcX blocker (Biolegend) for 30 min at room temperature using several combinations of the following fluorochrome-coupled antibodies: CD4-Alexa Fluor 488, CD25-BV421, CD45RA-PE/Cy7, CD8-PE/Dazzle-594, CD45RO-PerCP, CD62L-PE, CCR7-Alexa Fluor 700, CD57-BV785, CD21-APC/Fire-750, CD24-PerCP, CD73-BV711, CD11c-PE/Dazzle-594, IgM-PE, CD27-APC, CD38-Alexa Fluor 488, CD3-APC/Fire-750, IgD-Alexa Fluor 700, PD-1-APC, CD127-BV650, CD19-Pacific Blue, and CD138-BV605 (all from Biolegend). For the assessment of Th and Tc cell subsets, phorbol 12-myristate 13-acetate (50 ng/mL), ionomycin (1µg/mL), and monensin (4 µL/6 mL) were used for stimulating the PBMCs for 5 h at 37°C. The fluorochrome-coupled antibodies (IFN-γ-APC, IL-4-PE, and IL-17-BV421 [Biolegend]) were used for the detection of intracytoplasmic cytokines. According to the manufacturer’s instructions, fixation and permeabilization were performed using the Cytofix/Cytoperm fixation/permeabilization kit (BD Biosciences, Franklin Lakes, NJ, USA). A 4-laser LSR Fortessa flow cytometer (BD Biosciences) was used for sample acquisition.

Characterization of the following lymphocyte subsets was performed: CD4^+^ T cells (CD3^+^CD4^+^), naïve T cells (CD3^+^CD4^+^ or CD8^+^CD45RA^+^CD45RO^-^), CD8^+^ T cells (CD3^+^CD8^+^), memory T cells (CD3^+^CD4^+^ or CD8^+^CD45RA^-^CD45RO^+^), effector memory T cells (CD3^+^CD4^+^ or CD8^+^CD45RA^-^CD45RO^+^CD62L^-^CCR7^-^), central memory T cells (CD3^+^CD4^+^ or CD8^+^CD45RA^-^CD45RO^+^CD62L^+^CCR7^+^), exhausted T cells (CD3^+^CD4^+^CD8^+^PD-1^+/hi^), senescent T cells (CD3^+^CD4^+^CD8^+^CD57^+^), regulatory T cells (CD4^+^CD25^hi^CD127^lo/-^) and anergic T cells (CD3^+^CD4^+^CD8^+^CD73^+^) as depicted in **Supplementary Figure 1** and **Supplementary Figure 2**; Th1 (CD4^+^IFNγ^+^), Th2 (CD4^+^IL-4^+^), Th17 (CD4^+^IL-17^+^), Tc1 (CD8^+^IFNγ^+^), Tc2 (CD8^+^IL-4^+^) and Tc17 (CD8^+^IL-17^+^) as displayed in **Supplementary Figure 3**; total B cells (CD3^-^CD19^+^), CD21^-^ transitional B cells (CD19^+^CD27^-^CD38^hi^CD24^hi^CD21^-/lo^), CD21^+^ transitional B cells (CD19^+^CD27^-^CD38^hi^CD24^lo^CD21^+^), activated naïve B cells (CD19^+^CD27^-^IgD^+^CD38^-^CD24^-^CD11c^+^), resting naïve B cells (CD19^+^CD27^-^IgD^+^CD38^-^CD24^-^CD11c^-^), mature B cells (CD19^+^CD27^-^CD24^-/lo^), unswitched classical memory B cells (CD19^+^CD27^+^IgD^+^), plasmablasts (CD19^+^CD27^hi^ CD38^hi^), switched classical memory B cells (CD19^+^CD27^+^IgD^-^), non-classical CD27^-^IgD^-^ memory B cells (CD19^+^CD38^-/lo^CD24^+^CD27^-^IgD^-^), and non-classical CD27^-^IgD^+^ memory B cells (CD19^+^CD38^-/lo^CD24^+^CD27^-^IgD^+^) as depicted in **Supplementary Figure 4**.

PBMCs subset proportions at baseline were expressed as absolute numbers according to the number of lymphocytes in the complete blood count. The FlowJo v10.7 software (BD Biosciences) was used for the analyses.

### Assessment of cytokine/chemokine profiles

At baseline, 3 and 6 months after recruitment, twenty-nine serum cytokines and chemokines were measured using the MILLIPLEX Multi-Analyte Profiling Human Cytokine/Chemokine Magnetic Bead Panel 29-plex kit (EMD Millipore, Darmstadt, Germany) and a 2-laser Bio-Plex 200 suspension array system coupled to a Bio-Plex Pro Wash Station (Bio-Rad, Hercules, CA, USA), following the manufacturer’s instructions. Bead-fluorescence intensity readings for all the samples and standards were converted to the corresponding analyte concentrations using the Bio-Plex Manager software v6·2 (Bio-Rad).

The following analytes were measured: IL-1α, IL-1β, IL-1RA, IL-2, IL-3, IL-4, IL-5, IL-6, IL-7, IL-8, IL-10, IL-12p40, IL-12p70, IL-13, IL-15, IL-17A, IFNα2, IFNγ, tumor necrosis factor-α (TNF-α), TNF-β, macrophage inflammatory protein-1α/CCL3, monocyte chemoattractant protein 1/C–C motif chemokine ligand (CCL) 2, macrophage inflammatory protein-1β/CCL4, IP-10/C-X-C motif chemokine ligand 10, granulocyte-macrophage colony-stimulating factor, eotaxin-1/CCL11, granulocyte colony-stimulating factor, epidermal growth factor, and vascular endothelial growth factor.

### Assessment of circulating NETs

As previously described, serum NETs levels were assessed using a neutrophil elastase enzyme-linked immunosorbent assay (ELISA) (17) at baseline and 3 months after recruitment. Briefly, mouse anti-human neutrophil elastase (Calbiochem, Darmstadt, Germany) was diluted 1:2000 in coating buffer from the cell death detection ELISA kit (Roche, Basil, Switzerland) and incubated in high-binding 96-well plates overnight at 4 °C to generate mouse anti-human neutrophil elastase-coated plates. Plates were washed three times with PBS/Tween20 and then blocked with 1% bovine serum albumin (BSA) in PBS for 6 h at room temperature. Afterward, serum samples (1:10 in 1% BSA) were added to the plates and incubated overnight at 4 °C. Following three washes with PBS/Tween20 solution, the samples were incubated with anti-human DNA-peroxidase antibody (1:10 dilution in incubation buffer) from the cell death detection ELISA kit (Roche) for 1 h at room temperature. Next, the plates were washed five times with PBS/Tween20 before adding TMB substrate (Thermofisher Scientific, Waltham, MA, USA), followed by a stop solution. The plates were read at 450 nm, and the optic density index was calculated as previously described (17) in a Sunrise-RC/ST Evolyzer Plate Reader (Tecan Life Sciences, Männendorf, Switzerland).

### Assessment of anti-cellular (AC) antibodies

At baseline 3 and 6 months after recruitment, AC/antinuclear IgG antibodies were detected by indirect immunofluorescence using the HEp-2 cell line as substrate and the NOVA Lite HEp-2 ANA kit, according to the manufacturer’s instructions (INOVA Diagnostics, San Diego, CA, USA). Serum samples were tested at a 1:40 dilution using a Bee Line System (HTZ, East Grinstead, West Sussex, UK). Three experts read data for all samples, and the results were discussed and registered (AutoCyte Image Titer software, Burlington, NC, USA).

### Assessment of anti-SARS-CoV-2 IgG antibodies

According to the manufacturer’s instructions, anti-spike S1 domain antibodies were measured using an anti-SARS-CoV-2 ELISA IgG Kit (EUROIMMUN; Lübeck, Germany). The plates were processed using a DSX System (DYNEX Technologies, Chantilly, VA, USA). We calculated the cutoff value to be <0.51 AU at the 99th percentile using serum samples from our healthy donor bank that were collected from 2017 to 2018. Using this cutoff, we observed a sensitivity of 98.2%, a specificity of 98.9%, and an area under the curve (AUC) of 0.99. The cutoff value suggested by the manufacturer is ≥1.1 AU.

### Statistical analysis

We reported quantitative variables as medians and interquartile range (IQR). Medians were compared with Kruskal–Wallis and Wilcoxon tests. An iterative imputation method based on random forest was performed for missing data. For the assessment of the clinical and immunological features associated with the development of a sustained humoral immune response, we performed a univariate generalized linear model with binomial error using the variables at baseline, 3, and 6 months after recruitment. In this assessment, we performed an analysis for each one of the time-points. Since the most important factor for the development of a sustained humoral immune response is the COVID-19 severity, the model included this feature as a two-level fixed factor (critical vs non-critical). We included the significant variables in a multivariate generalized linear model and performed stepwise selection using minimum Akaike information criteria. Odds ratios (OR) with 95% confidence intervals (95% CI) were calculated. Finally, to address the effect of the changes of the serum levels of cytokines and chemokines throughout the follow-up in the development of a sustained humoral immune response, we performed a univariate generalized linear mixed model with binomial error. *Post-hoc* comparisons were performed for significant variables at 6 months after recruitment in the following group of patients: critical COVID-19 with and without sustained humoral immune response and non-critical COVID-19 with and without this primary outcome. Statistical analysis was performed using the R project software (R Core Team (2021, R: A language and environment for statistical computing. R Foundation for Statistical Computing, Vienna, Austria. URL http://www.R-project.org/).

## Results

Of the 103 patients in this study, 56 (54.3%) were women. The cohort’s median (IQR) of age was 50.0 (41.5–58.0) years. The most frequent comorbidities were obesity, hypertension, and type 2 diabetes, which were reported in 48 (46.2%), 28 (26.9%), and 24 (23.1%) patients, respectively. At baseline, 46 (44.2%) patients had mild/moderate disease, 31 (29.8%) had severe disease, and 27 (25.9%) had critical disease. The baseline clinical and laboratory features of patients with COVID-19 according to disease severity are shown in **Tables 1** and **2**. Similar to previous COVID-19 cohorts, patients with critical disease were older and had a higher body mass index (**Table 1**). Likewise, patients with severe and critical COVID-19 had many markers of adverse prognosis including neutrophilia, lymphopenia, and increased D-dimer (**Table 2**).

**Table 1:** Clinical characteristics of patients with mild to critical COVID-19 at recruitment.

Variable	Mild/moderate n = 46	Severe n = 30	Critical n = 27	P-value
Female, n (%)	27 (48.21)	19 (33.92)	10 (17.85)	
Male, n (%)	19 (40.42)	11 (23.40)	17 (38.17)	
Age (years)	47.50 (31.75–55.00)	52.00 (43.50–47.25)	58.00 (44.00–64.00)	0.023
Number of symptoms	4.50 (3.00–6.00)	4.80 (4.00–6.00)	4.00 (2.50–5.00)	0.035
Body mass index (kg/m^2^)	27.00 (24.25–31.00)	29.50 (27.25–34.00)	31.00 (29.00–33.00)	<0.001
SpO_2_ (%)	94.00 (93.00–96.00)	93.5 (90.25–94.75)	92.86 (91.50–95.00)	0.257
Systolic blood pressure (mmHg)	121.00 (113.00-129.5)	118.00 (108.00-120.50)	120.00 (115.00-129.00)	0.115
Diastolic blood pressure (mmHg)	76.00(64.00-96.00)	74.00 (64.75-79.25)	80.00 (70.00-86.00)	0.122
Mean arterial pressure (mmHg)	90.67 (82.50-96.17)	86.33 (80.50-92.75)	91.67 (88.00-97.00)	0.071
Respiratory rate (breaths/min)	20.00 (18.00-22.00)	20.00 (19.00-21.25)	20.50 (19.00-25.35)	0.160
Temperature (°C)	36.50 (36.30-36.60)	36.45 (36.30-36.60)	36.40 (36.20-36.60)	0.648
Heart rate (beats/min)	80.00 (66.00-93.50)	88.00 (82.75-96.00)	81.00 (75.00-91.00)	0.045

All values are expressed as median (interquartile range) unless otherwise specified.

SpO_2_, oxygen saturation.

**Table 2:** Laboratory characteristics of patients with mild to critical COVID-19 at baseline.

Variable	Mild/moderaten=46	Severen=30	Criticaln=27	P-value
Leukocytes (cells/mm^3^)	6000.00 (4625.00–7750.00)	7250.00 (5050.00–8775.00)	7900.00 (7100.00–10750.00)	<0.001
Total lymphocytes (cells/mm^3^)	1714.20 (1348.80–2371.70)	965.80 (759.70–1410.80)	737.00 (547.80–942.60)	<0.001
Total lymphocytes (%)	31.45 (21.93–39.95)	17.15 (10.12–26.60)	8.10 (5.65–11.95)	<0.001
Total neutrophils (cells/mm^3^)	3321.00 (2536.00–4244.00)	5700.00 (3750.00–7003.00)	6975.00 (6049.00–9419.00)	<0.001
Total neutrophils (%)	55.95 (50.35–65.00)	77.05 (64.05–84.55)	86.80 (81.40–90.95)	<0.001
Total monocytes (cells/mm^3^)	477.60 (387.90–607.20)	337.40 (261.80–472.60)	318.00 (227.00–518.00)	0.033
Total monocytes (%)	8.15 (6.52–10.15)	5.20 (3.27–7.87)	4.00 (2.70–6.50)	<0.001
N/L ratio	1.71 (1.28–3.00)	4.43 (2.41–8.49)	10.81 (7.21–16.35)	<0.001
Haemoglobin (g/dL)	14.25 (13.30–15.28)	14.25 (12.85–14.88)	14.00 (13.35–15.05)	0.892
Haematocrit (%)	42.30 (40.50–45.50)	43.20 (39.00–45.05)	42.80 (40.30–45.60)	0.732
Platelets (cells/mm^3^)	272.50 (204.00–362.50)	267.60 (182.00–348.20)	306.00 (236.00–362.50)	0.248
Glucose (mg/dL)	123.64 (95.75–162.12)	129.70 (93.25–149.00)	123.00 (110.00–134.00)	0.889
HbA1c (%)	7.04 (6.22–7.70)	7.01 (6.02–7.65)	6.70 (5.45–7.13)	0.040
ALP (U/L)	85.70 (76.46–98.18)	85.84 (72.25–99.21)	73.00 (58.00–89.50)	0.018
AST (U/L)	32.48 (25.25–42.36)	35.00 (19.00–51.49)	31.00 (19.00–42.71)	0.390
Albumin (g/dL)	3.82 (3.66–4.07)	3.79 (3.57–4.00)	3.60 (3.39–4.15)	0.123
Creatinine (g/dL)	0.80 (0.70–1.08)	1.22 (0.80–1.31)	0.90 (0.65–1.09)	0.643
CRP (mg/dL)	40.77 (10.38–69.69)	39.30 (4.20–73.05)	32.00 (2.90–103.33)	0.718
Ferritin (ng/dL)	262.60 (117.80–490.80)	382.10 (130.40–574.70)	405.30 (198.90–756.60)	0.166
CPK (U/L)	53.05 (33.50–63.46)	43.50 (28.50–54.19)	34.00 (23.00–52.00)	0.034
D-dimer (ng/mL)	230.00 (189.00–345.00)	320.00 (190.00–492.50)	530.00 (350.00–920.00)	<0.001
PAFI ratio	312.00 (280.50–361.50)	273.00 (209.00–310.00)	248.00 (187.00–298.00)	0.001

All values are expressed as median (interquartile range) unless otherwise specified. ALP, alkaline phosphatase; AST, aspartate aminotransferase; CPK, creatine phosphokinase; CRP, C-reactive protein; HbA1c, A1c Hemoglobin; N/L ratio, neutrophil/lymphocyte ratio; PAFI ratio, PaO_2_/FiO_2_; SpO_2_, oxygen saturation.

**Figure 1:**
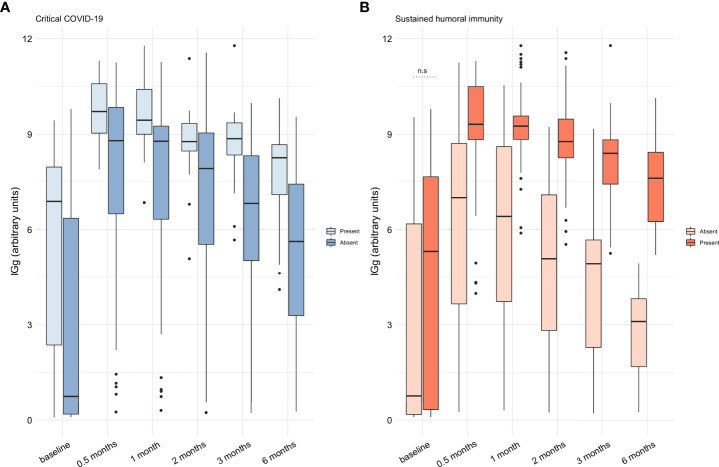
Assessment of the anti-SARS-CoV-2 IgG throughout time in the cohort of patients from mild to critical COVID-19. **(A)** The titers of anti-SARS-CoV-2 IgG was higher at all timepoints in patients with critical COVID-19 (P<0.05). **(B)** Patients with a sustained humoral immune response had a higher amount of anti-SARS-CoV-2 IgG at all timepoints (P<0.05) but not at baseline (n.s.). Anti-SARS-CoV-2 IgG titers are expressed as medians with interquartile range and were compared with the Wilcoxon test. AU: arbitrary units; IgG: immunoglobin G; SARS-CoV-2: severe acute respiratory syndrome coronavirus 2. n.s: non-significant.

Using our calculated cutoff value, 67 patients (65%) had positive anti-SARS-CoV-2 IgGs at baseline. Fifty-eight (56.3%) were positive for anti-SARS-CoV-2 antibodies at baseline using the cutoff value recommended by the manufacturer (≥1.1 AU). The positivity for anti-SARS-CoV-2 antibodies remained over 95% in the following time points regardless of the used cutoff point. Afterward, we aimed to evaluate the proportion of patients maintaining a virus-neutralizing humoral response as described in our primary outcome. Sixty-eight (66%) patients had a sustained humoral immune response, which was more frequent in subjects with critical disease in comparison to those with non-critical COVID-19 (88% vs 57%, respectively, P=0.004). In **Figure 1** we show the titers and kinetics of the anti-SARS-CoV-2 IgG according to disease severity and the development of a sustained humoral immune response. Patients with critical COVID-19 and a sustained humoral response were characterized by a higher amount of anti-SARS-CoV-2 IgG, but the amount of anti-SARS-CoV-2 IgG at baseline was not different in patients with and without sustained humoral immunity.

**Figure 2:**
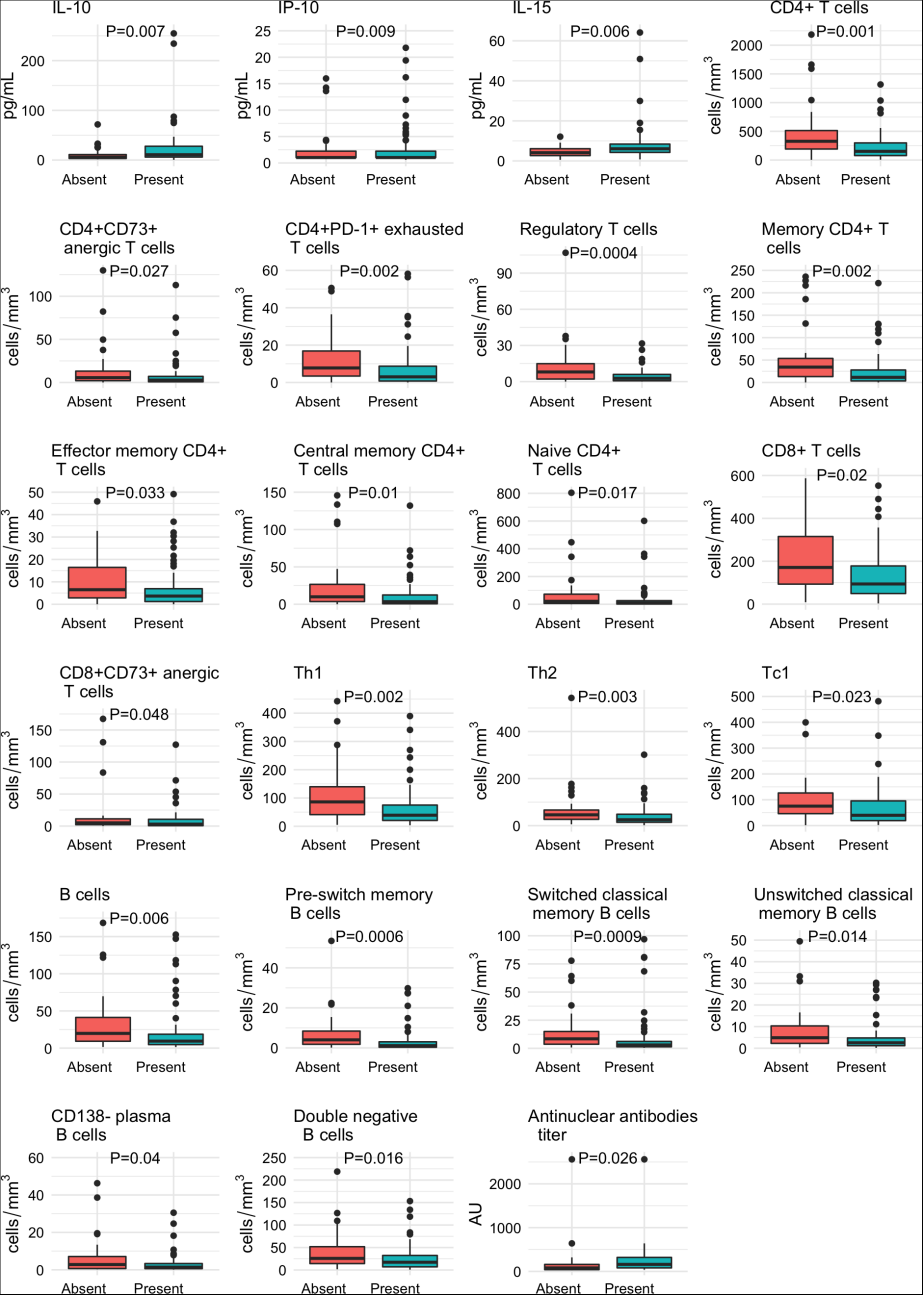
Baseline differences in the immunological profile of patients with COVID-19 according to the development of a sustained immune humoral response. The absolute numbers of T and B cell subsets, the cytokines, chemokines and AC/ANA titers are expressed as medians and interquartile ranges and were compared with the Wilcoxon test.

In **Supplementary Table 1**, we depict the comparison of the immunological features at baseline between patients who developed the primary outcome and those who did not. As shown in the **Supplementary Table 1** and in accordance with their more frequent critical status and more pronounced lymphopenia, patients with sustained humoral immune response were characterized by a lower proportion of many CD4+ T cell subsets, CD8+ lymphocytes and B cell subtypes. The immunological features that were different between patients with and without a sustained humoral immune response at baseline and that achieve statistically significance are described in **Figure 2**.

**Figure 3:**
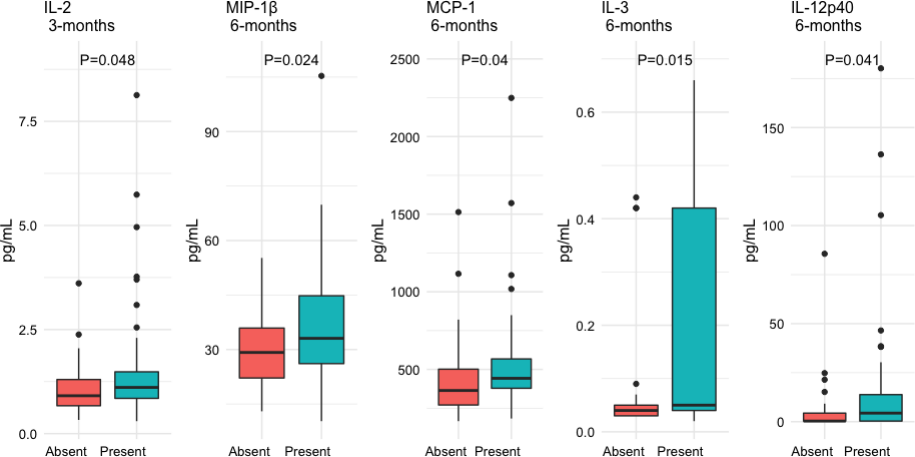
Differential expression of serum cytokine and chemokines 3 and 6 months after recruitment of patients with COVID-19 according to the presence of a sustained immune humoral response. The serum levels of the cytokines and chemokines are expressed as medians and interquartile ranges and were compared with the Wilcoxon test.

In the **Supplementary Tables 2**, **3** we show the comparison of the variables 3 and 6 months after recruitment between patients who developed a sustained humoral immune response and those who did not. In **Figure 3**, we depict the variables that achieve a statistically significant difference between patients with and without a sustained humoral immune response during follow-up. Three months after recruitment, patients with a sustained humoral immune response had higher levels of IL-2, whilst at 6 months they had increased serum levels of MIP-1β, MCP-1, IL-3 and IL-12p40 (**Figure 3**).

Using the development of a sustained immune humoral response as an outcome variable, we performed a univariate analysis with the variables that were available at baseline, 3 and 6 months after recruitment. The results obtained from this analysis are depicted in the **Supplementary Table 4**. In the multivariate analysis, the features independently associated with a sustained humoral immune response at baseline were the following: critical disease (OR 58.823 [95% CI 4.480–1666.66], P<0.0001), absolute number of lymphocytes (cells/mm^3^) (OR 1.001 [95% CI 1.0001–1.002], P=0.025), serum IP-10 (pg/mL) (OR 1.001 [95% CI 1.0003–1.002], P<0.0001), IL-4 (pg/mL) (OR 0.997 [95% CI 0.995–0.999], P=0.001), IL-2 (pg/mL) (OR 1.358 [95% CI 1.010–3.553], P=0.038), absolute number of regulatory T cells (cells/mm^3^) (OR 0.913 [0.826–0.991], P=0.025), absolute number of CD8^+^ T cells (cells/mm^3^) (OR 0.996 [95% CI 0.993–0.999], P=0.038), and positive AC antibodies (OR 14.459 [95% CI 2.645–147.395], P<0.001).

**Figure 4:**
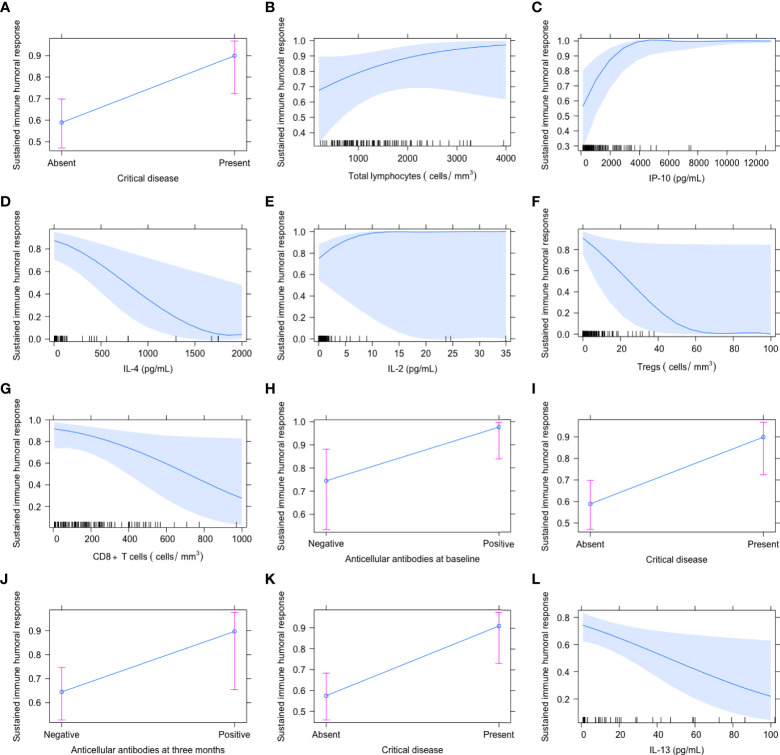
Independent variables associated with the presence of a sustained immune humoral response at baseline **(A–H)**, 3 **(I, J)** and 6 months **(K, L)** after COVID-19 onset. The proportion of patients with a sustained immune humoral response was defined as those maintaining anti-SARS-CoV-2 IgG titers above 4.99, which has been shown to correlate with a viral neutralizing capacity. The graphs represent the effect of the explanatory variables on the probability to have a sustained immune humoral response, being the maximum probability 1 (100%).

Three months after recruitment, the features independently associated with a sustained humoral immune response were the diagnosis of critical disease (OR 6.944 [95% CI 2.008–35.714], P=0.001) and the positivity of AC antibodies (OR 10.975 [95% CI 1.855–192.629], P=0.005). The presence of critical COVID-19 (OR 6.666 [95% CI 1.886–34.481], P=0.002) and serum levels of IL-13 (pg/mL) OR 0.978 [95% CI 0.957-0.997], P=0.028) were the explanatory features of a sustained humoral immune response 6 months after recruitment. The effect of the explanatory variables at each timepoint on the possibility to develop a sustained immune humoral response is depicted in **Figure 4**. As shown in the graphs, patients with a higher possibility to develop a sustained humoral immune response (close to 1 or 100%) were those with critical disease, positive AC antibodies, higher amounts of IL-2, IP-10 and of total lymphocytes, and decreased IL-4, Tregs, CD8+ T cells, and IL-13.

Finally, we aimed to assess if the changes of the cytokines and chemokines throughout time were related to the development of the sustained immune humoral response. With the univariate generalized linear mixed model, we observed that the changes in the serum levels of 5 cytokines (TNF-β, IL-5, IL-3, IL-1α and IL-13) during follow-up were associated with the development of a sustained immune humoral response. To include the effect of the disease severity in the analysis, we assessed the serum levels of these cytokines 6 months after recruitment among the following comparison groups: critical and non-critical COVID-19 patients with and without the presence of a sustained humoral immune response. The findings of this analysis are presented in **Supplementary Table 5**. As shown in **Supplementary Figure 5**, the main differences in cytokine levels were found in critical patients without a sustained humoral immune response. This patient group showed the highest serum cytokine levels compared with critical patients with a sustained humoral response and both groups of non-critical patients. However, patients with critical disease and sustained humoral immune response had higher levels of IL-3 compared with the other groups.

## Discussion

In this study, we stablished associations between the clinical and immunological features during acute COVID-19 and the development of a sustained humoral immune response. Also, with the longitudinal follow-up, we were able to describe not only the kinetics of the anti-SARS-CoV-2 IgGs, but also the changes in the serum cytokines and chemokines related to this outcome. In our study, we found that critical COVID-19 is a key factor associated with the development of a sustained immune humoral response with anti-SARS-CoV-2 IgG titers that correlate with a viral neutralizing capacity. At baseline and in the prospective analysis, the diagnosis of critical COVID-19 was the main factor associated with a sustained humoral immune response. In addition, this response was related to lower serum levels of IL-4, IL-13 and a decreased proportion of CD8^+^ and regulatory T cells, a higher absolute number of total lymphocytes and increased serum concentrations of IP-10, IL-2, and AC antibodies.

Zhang, et al. described that SARS-CoV-2 neutralizing antibodies could persist in 49% of patients after disease onset and are positively associated with disease severity (18). Also, patients who survive severe COVID-19 have a faster and more robust humoral response compared with patients with less severe disease and develop higher titers of anti-SARS-CoV-2 antibodies (19), which agrees with our findings.

We observed that an immune profile associated with critical COVID-19 is a fundamental driver of the development of a sustained humoral immune response. In this regard, an effective T cell response is a key prognostic factor in COVID-19. CD4^+^ and CD8^+^ lymphopenia are hallmarks of severe COVID-19 (20). The expression of genes related to apoptosis has been observed in memory T cells from patients with COVID-19 (20). One theory is that these memory cells experience activation-induced cell death, which is difficult to overcome in the context of lymphopenia (20).This is in line with our results since we found that patients with severe COVID-19 had a lower proportion of memory CD4^+^ T cells and of total lymphocytes. A dysfunction in the cytotoxic response and the production of IFN-γ in CD8^+^ T cells has been demonstrated in severe COVID-19 (21). Our results coincide with other reports, which state that during severe infection, most patients present lower amounts of CD8^+^ T cells. CD8^+^ T cell lymphopenia is especially prominent in patients with prolonged disease courses (22). Previous studies have demonstrated that an effective CD8^+^ T cells response is the key factor for the resolution of the SARS-CoV-2 infection (23). Therefore, the CD8^+^ T cell lymphopenia may be the reflection of the progression to a critical disease state, which was the main factor associated to the development of a sustained humoral immune response in our study.

In this work, we found a direct relationship between the amount of lymphocytes and the development of a sustained humoral immune response. Although lymphopenia is a characteristic feature of severe COVID-19, previous studies have shown that an effective humoral response to vaccines is related to a higher percentage of B lymphocytes in multiple myeloma patients (24). Likewise, subjects receiving B cell depletion therapies are less likely to have a humoral response after SARS-CoV-2 vaccination (25). Also, it has been shown that hospitalized patients with COVID-19 have a higher amount of follicular T helper cells, which directly correlates with anti-SARS-CoV-2 antibodies and is inversely correlated with the proportion of regulatory T cells (26). Therefore, even in the context of lymphopenia, a high number of certain lymphocyte subsets may be necessary for the development of a sustained humoral immune response.

Following viral antigen-mediated lymphocyte stimulation, there is a robust production of IFN-γ, TNF-α, and IL-2 in COVID-19 (27). A previous study found that this potent CD4^+^ response correlates with serum anti-SARS-CoV-2 IgGs (28), consistent with our results since we found that IL-2 is a determinant of the sustained humoral immune response. In this regard, this prominent inflammatory response may not be counteracted by the low levels of regulatory T cells, which we found to be inversely related to sustained humoral immunity. Previous work has suggested that the decrement in regulatory T cells in patients with severe COVID-19 is the result of their re-distribution into the lung, since animal models have proven that these cells are fundamental for the resolution of acute respiratory distress syndrome (ARDS) (20). Other theories to explain the decreased amount of this cell subset in COVID-19 are the IL-6 induced transformation of regulatory T cells into Th17 effector cells and the hypoxia-induced degradation of Foxp3 through the activation of the hypoxia-inducible factor-1α (29). This prior evidence supports our findings. Besides, regulatory T cells are fundamental to prevent immune system hyperactivation, including autoimmunity (29), which may be related to our finding of a correlation between AC antibodies and a sustained immune humoral response. The production of AC antibodies could be caused by infection-induced clonal expansion and the infiltration of self-reactive cells, leading to the development and activation of lymphoid follicles, favoring a broad and robust humoral response that may include the production of autoantibodies, as described in other viral infection, such as Epstein-Barr virus (30).

Critical COVID-19 patients also present IP-10 driven extrafollicular humoral responses, which are strongly correlated with large antibody-secreting cells expansion and early production of high concentrations of SARS-CoV-2-specific neutralizing antibodies (31). IP-10 and IL-2 are biomarkers of disease severity (32), which supports our findings. Also, extrafollicular B cell responses are related to a higher production of anti-SARS-CoV-2 autoantibodies and could cause autoimmunity because of the lack of certain immune checkpoints that help avoid the production of autoantibodies (33). Previous studies have found a correlation between the production of autoantibodies and anti-SARS-CoV-2 IgGs in patients with critical disease. Anti-chromatin antibodies are a sign of severe and critical COVID-19 and a sign of adverse prognosis (34), which agrees with our findings.

During the longitudinal follow-up, we observed that changes in growth factors, chemokines, and interleukins were associated with the presence of a sustained immune humoral response in COVID-19. Serum levels of IL-4 and IL-13 were negatively correlated with the robust inflammatory response and CD8^+^ T cell counts. Although hyperinflammation is a hallmark of critical COVID-19, previous research has shown that this leads to a lower immune response to subsequent stimuli (35). In line with these previous findings, the longitudinal cytokine behavior observed in our study showed that most patients with a sustained humoral immune response had lower levels of cytokines at the end of follow-up, despite being in a critical stage of the disease at baseline, with a peak of IL-3 and IL-2 at 3 months post recruitment. Only the increased levels of IL-3 persisted at 6 months after baseline in critical patients, whereas patients with higher concentrations of cytokines had no sustained humoral immune response. In this regard, we hypothesize that the lower amount of cytokines in patients with sustained humoral immune response who were previously critically-ill, may be the result of a higher threshold in the capacity to respond to an immune stimuli, but further studies are needed to confirm this theory. Because IL-3 is a key factor in activating B cells and inflammatory disease (36) we conclude that exceptionally high levels of IL-3 are directly related to disease severity and humoral immune response.

The role of IL-4 in COVID-19 severity is controversial. Consistent with the current findings, several previous studies have demonstrated that serum IL-4 levels are not increased in critical COVID-19 patients (37) however, contradictory evidence also exists. IL-4 is a regulatory cytokine that limits the inflammatory response against microorganisms (38). Moreover, IL-4 regulates the secretion of IL-1, TNF-β, and prostaglandin E_2;_ therefore, lower serum IL-4 concentrations may favor a higher production of these mediators, which are known to promote tissue damage. IL-4 also limits autoimmune responses (38) consistent with our finding of a lower IL-4 concentration and positive AC antibodies among patients with a sustained humoral immune response. Other studies have found a negative correlation between IL-4 and serum levels of anti-SARS-CoV-2 antibodies (r=−0.208, P=0·023) (39) which agrees with our results.

The main limitation of our study is its observational design, which only allow us to establish associations without inferring any causality. In this regard, we acknowledge that the interpretation of our results is based in the extensive research regarding the COVID-19 immune response and not in a functional or experimental analysis of the features that we found to be associated with the sustained humoral immune response. Furthermore, we only included Mexican-mestizo patients, and we did not assess the viral load and SARS-CoV-2 genotyping, which were unavailable when the study was conducted. Additionally, we could not address the neutralizing capacity of anti-SARS-CoV-2 specific antibodies. Nonetheless, in the clinical practice, the assessment of the CD8^+^ and regulatory T cells as well as the serum levels of IL-4 and IP-10 can be used to identify patients with a more robust humoral immunity allowing us to address the clinical significance of these antibodies in the appearance of post-COVID-19 syndrome and as markers of a sustained humoral immunity.

## Conclusion

A sustained humoral immune response against SARS-CoV-2 was associated with severe disease and quantitative alterations in the T cell subsets, cytokines and chemokines as well as with the production of AC antibodies. It is possible that a defective cellular anti-viral response leads to a more prolonged and severe disease with a higher antigen exposure and a more robust production of antibodies. A low amount of regulatory T cells and IL-4 may not be able to counteract this hyperinflammation, which may allow the B cells to produce a high quantity of IgGs, some of them against self-antigens which is clinically manifested as the presence of AC antibodies. Eventually, this hyperstimulation of the immune system may result in a higher activation threshold in the long-term, which may explain the finding of a lower amount of cytokines in patients with a sustained immune humoral response who were previously critically-ill.

## Data availability statement

The original contributions presented in the study are included in the article/**Supplementary Material**. Further inquiries can be directed to the corresponding authors.

## Ethics statement

The studies involving human participants were reviewed and approved by Instituto Nacional de Ciencias Médicas y Nutrición Salvador Zubirán (Ref. 3341). The patients/participants provided their written informed consent to participate in this study.

## Author contributions

JT-R, JL-G, AV-C, JL-L, AP-F, RT-C, MN-A, BA-C, AA-A, JM-M, NM-D, CN-A, MR-G, LL, SR-R, VS-H, RC-D, GJ-V, DM-S, LM-J, RV-V, JF-L, and DG-M participated in the conceptualization of the study. JT-R, JL-G, AV-C, AP-F, JM-M, NM-D, CN-A, MR-G, LL, LM-J, LM-J, LL-L, JN-T, JF-L, RV-V, HG-R, and DG-M collaborated with the study design. Data acquisition and analysis were done by JT-R, JL-G, AV-C, DG-M, JL-L, AP-F, RT-C, MN-A, BA-C, AA-A, JM-M, NM-D, CN-A, GJ-V, DM-S, LL-L, and JN-T. Data interpretation was done by JT-R, JL-G, AV-C, DG-M, JL-L, AP-F, RT-C, MN-A, BA-C, AA-A, JM-M, NM-D, CN-A, MR-G, LL, SR-R, VS-H, RC-D, GJ-V, DM-S, LM-J, LM-J, JF-L, RV-V, and DG-M. JT-R, JL-G, AV-C, DG-M, JL-L, AP-F, RT-C, MN-A, BA-C, AA-A, JM-M, NM-D, CN-A, MR-G, LL, SR-R, VS-H, RC-D, GJ-V, DM-S, LM-J, LM-J, and RV-V wrote the original draft. All authors contributed to the article and approved the submitted version.

## Funding

The funds provided by Fundación Carlos Slim were only used to purchase reagents and other consumables. Fundación Carlos Slim employees were involved in the conduct of the study and contributed to the collection and interpretation of the data. The funder did not have any role in the writing or decision to submit the manuscript for publication. The authors worked independently of the funder and all authors, external and internal, had full access to all the data in the study and take responsibility for the integrity of the data and the accuracy of the data analysis.

## Acknowledgments

The authors would like to acknowledge the technical support provided by the Red de Apoyo a la Investigación (RAI). The graphical abstract was created using the Biorender website.

## Conflict of interest

The authors declare that the research was conducted in the absence of any commercial or financial relationships that could be construed as a potential conflict of interest.

## Publisher’s note

All claims expressed in this article are solely those of the authors and do not necessarily represent those of their affiliated organizations, or those of the publisher, the editors and the reviewers. Any product that may be evaluated in this article, or claim that may be made by its manufacturer, is not guaranteed or endorsed by the publisher.

## Supplementary material

Supplementary Figure 1 Gating strategy for the evaluation of T helper, cytotoxic, naïve, exhausted, anergic, regulatory, senescent cell subsets.

Supplementary Figure 2 Gating strategy for the evaluation of T memory cell subsets.

Supplementary Figure 3 Gating strategy for the evaluation of CD4^+^ Th1, Th2, Th17 and CD8^+^ Th1, Th2, Th17 cell subsets

Supplementary Figure 4 Gating strategy for the evaluation of B cell subsets

Supplementary Figure 5 Repeated-measures analysis of serum cytokines by disease severity and development of sustained humoral immune response. **(A)** TNF-β. **(B)** IL-5. **(C)** IL-3. **(D)** IL-1α. **(E)** IL-13. The significant *post-hoc* comparisons are shown. IL: interleukin; SHIR: sustained humoral immune response; TNF-β: tumor necrosis factor-β.

Supplementary Table 1 Baseline immunological features of patients with COVID-19 according to the presence of a sustained immune humoral response. The absolute numbers of T and B cell subsets were compared with the Wilcoxon test.

Supplementary Table 2 Evaluation of the serum levels of cytokines, chemokines, NETs and AC antibodies 3 months after recruitment according to the development of a sustained humoral immune response. The serum levels of the cytokines, chemokines, NETs and the titers of AC antibodies are expressed as medians and interquartile ranges and were compared with the Wilcoxon test.

Supplementary Table 3 Comparison of the serum levels of cytokines, chemokines and AC antibodies in patients with COVID-19, 6 months after recruitment according to the diagnosis of a sustained humoral immune response. The serum levels of the cytokines and chemokines are expressed as medians and interquartile ranges and were compared with the Wilcoxon test.

Supplementary Table 4 Univariate logistic regression analysis of the statistically significant features associated to the development of a sustained humoral immune response in patients with COVID-19.

Supplementary Table 5 Repeated-measures analysis of the differential serum levels of cytokines 6 months after recruitment considering the disease severity and the development of a sustained humoral immune response.
