# Supplementary material for: Clinical and immunological features associated to the development of a sustained immune humoral response in COVID-19 patients: Results from a cohort study
Source: Front Immunol. 2022 Aug 15;13:943563. doi: 10.3389/fimmu.2022.943563 (PMC9421299; doi:10.3389/fimmu.2022.943563)

**Supplementary Table 1.** Baseline immunological features of patients with COVID-19 according to the presence of a sustained immune humoral response. The absolute numbers of T and B cell subsets were compared with the Wilcoxon test.

| **Variable** | **No sustained humoral immune response**  **Median (IQR)** | **Sustained humoral immune response**  **Median (IQR)** | **P-value** |
| --- | --- | --- | --- |
| **CD4^+^ T cells (cells/mm^3^)** | **326.74 (192.10-513.39)** | **151.86 (77.91-298.37)** | **0.001** |
| CD4^+^CD57^+^ T cells (cells/ mm^3^) | 2.09 (0.64-6.72) | 1.55 (0.39-3.67) | 0.224 |
| MFI of CD57 in CD4^+^ T cells | 21155 (10432-33820) | 25169 (14387-37236) | 0.378 |
| **CD4^+^CD73^+^ T cells (cells/mm^3^)** | **5.57 (2.05-13.15)** | **2.75 (0.77-7.05)** | **0.027** |
| MFI of CD73 in CD4^+^ T cells | 3128 (2928-3780) | 2998 (2583-3734) | 0.188 |
| **CD4^+^PD-1^+^ T cells (cells/mm^3^)** | **7.77 (3.43-16.85)** | **3.00 (0.77-8.71)** | **0.002** |
| MFI of PD-1 in CD4^+^ T cells | 2335 (2038-2494) | 2398 (2085-2752) | 0.374 |
| **Regulatory T cells (cells/mm^3^)** | **8.01 (2.12-14.87)** | **2.72 (0.68-5.98)** | **<0.01** |
| **Memory CD4^+^ T cells (cells/mm^3^)** | **34.31 (13.12-53.65)** | **11.41 (3.39-27.85)** | **0.002** |
| **Effector memory CD4^+^ T cells (cells/mm^3^)** | **6.50 (2.85-16.45)** | **3.66 (1.21-6.94)** | **0.033** |
| **Central memory CD4^+^ T cells (cells/mm^3^)** | **9.95 (3.60-26.63)** | **3.43 (0.68-12.43)** | **0.010** |
| **Naïve CD4^+^ T cells (cells/mm^3^)** | **21.31 (6.09-72.63)** | **10.41 (1.63-25.98)** | **0.017** |
| **CD8^+^ T cells (cells/mm^3^)** | **171.03 (93.23-315.34)** | **93.97 (49.30-178.06)** | **0.020** |
| CD8^+^CD57^+^ T cells (cells/mm^3^) | 4.97 (2.17-11.25) | 3.05 (0.63-10.56) | 0.182 |
| MFI of CD57 on CD8^+^ T cells | 29948 (25364-39460) | 29580 (19838-40340) | 0.618 |
| CD8^+^PD-1^+^ T cells (cells/mm^3^) | 3.18 (1.18-7.00) | 2.37 (0.52-5.51) | 0.294 |
| MFI of PD-1 in CD8^+^ T cells | 1997 (1784-2248) | 2008 (1758-2384) | 0.889 |
| **CD8^+^CD73^+^ T cells (cells/mm^3^)** | **4.43 (1.32-10.59)** | **2.09 (0.64-5.39)** | **0.048** |
| MFI of CD73 in CD8^+^ T cells | 3758 (3353-4037) | 4102 (3276-4728) | 0.121 |
| Memory CD8^+^ T cells (cells/mm^3^) | 0.47 (0.11-1.10) | 0.40 (0.10-1.27) | 0.930 |
| Effector memory CD8^+^ T cells (cells/mm^3^) | 0.27 (0.04-0.95) | 0.21 (0.05-0.84) | 0.704 |
| Central memory CD8^+^ T cells (cells/mm^3^) | 0.00 (0.00-0.06) | 0.00 (0.00-0.05) | 0.943 |
| Naïve CD8^+^ T cells (cells/mm^3^) | 9.89 (4.33-25.44) | 4.73 (2.02-18.25) | 0.114 |
| **Th1 (cells/mm^3^)** | **86.18 (41.15-139.77)** | **39.06 (20.59-74.98)** | **0.002** |
| **Th2 (cells/mm^3^)** | **45.96 (26.78-66.40)** | **25.15 (14.19-48.41)** | **0.003** |
| Th17 (cells/mm^3^) | 1.03 (0.59-2.54) | 0.92 (0.36-4.22) | 0.859 |
| **Tc1 (cells/mm^3^)** | **75.43 (46.25-126.30)** | **39.91 (19.28-95.62)** | **0.023** |
| Tc2 (cells/mm^3^) | 35.00 (22.02-64.53) | 25.79 (10.17-57.09) | 0.187 |
| Tc17 (cells/mm^3^) | 0.26 (0.09-0.66) | 0.22 (0.05-0.95) | 0.947 |
| B cells (cells/mm^3^) | 128.02 (75.94-292.77) | 103.32 (63.06-164.62) | 0.066 |
| **Memory B cells (cells/mm^3^)** | **19.79 (9.23-41.25)** | **9.40 (4.73-18.69)** | **0.006** |
| IgD memory B cells (cells/mm^3^) | 1.56 (0.73-2.58) | 1.20 (0.46-3.56) | 0.831 |
| **Pre-switch memory B cells (cells/mm^3^)** | **4.02 (1.79-8.40)** | **1.14 (0.34-3.05)** | **<0.001** |
| **Switched classical memory B cells (cells/ mm^3^)** | **8.43(3.64-14.96)** | **3.01 (1.31-6.13)** | **<0.0001** |
| **Unswitched classical memory B cells (cells/mm^3^)** | **4.85 (2.24-10.32)** | **2.52 (1.16-4.75)** | **0.014** |
| Plasmablasts (cells/mm^3^) | 3.48 (1.47-8.55) | 1.86 (1.07-5.28) | 0.062 |
| Plasma B cells (cells/mm^3^) | 0.79 (0.30-1.26) | 0.41 (0.23-0.82) | 0.096 |
| **CD138^-^ plasma B cells (cells/mm^3^)** | **2.81 (0.68-7.10)** | **1.42 (0.61-3.35)** | **0.040** |
| CD27^-^ B cells (cells/mm^3^) | 99.69 (61.05-220.49) | 87.75 (51.00-138.64) | 0.116 |
| T1^+^T2 B cells (cells/mm^3^) | 10.38 (5.17-21.53) | 8.84 (4.92-13.44) | 0.479 |
| Transitional CD21^-^ B cells (cells/mm^3^) | 2.28 (1.13-6.66) | 2.16 (1.21-5.62) | 0.826 |
| Transitional CD21^+^ B cells (cells/mm^3^) | 3.85 (2.71-12.12) | 4.78 (2.67-6.95) | 0.479 |
| CD24^+^CD38^lo/-^ B cells (cells/mm^3^) | 8.13 (2.65-11.70) | 4.93 (3.00-11.04) | 0.643 |
| IgD^-^ B cells (cells/mm^3^) | 1.57 (0.60-3.04) | 1.15 (0.40-3.13) | 0.623 |
| IgD^+^ B cells (cells/mm^3^) | 3.09 (1.06-5.95) | 2.65 (1.65-4.61) | 0.777 |
| Mature B cells (cells/mm^3^) | 81.63 (48.20-187.91) | 68.56 (31.95-104.20) | 0.068 |
| Naïve B cells (cells/mm^3^) | 54.08 (31.30-100.83) | 39.62 (24.34-75.34) | 0.133 |
| Resting naïve B cells (cells/mm^3^) | 5.27 (2.41-9.75) | 6.97 (3.11-20.96) | 0.160 |
| **Activated naïve B cells (cells/mm^3^)** | **51.51 (26.45-89.95)** | **25.38 (3.96-59.51)** | **0.005** |
| **Total Double negative B cells (cells/mm^3^)** | **25.85 (14.35-51.48)** | **17.26 (7.04-32.40)** | **0.016** |
| **Double negative 1 B cells (cells/mm^3^)** | **7.27 (3.78-15.42)** | **4.96 (2.07-10.27)** | **0.027** |
| **Double negative 2 B cells (cells/mm^3^)** | **2.98 (1.19-5.59)** | **1.56 (0.82-4.23)** | **0.044** |
| **Double negative 3 B cells (cells/mm^3^)** | **11.95 (7.36-23.72)** | **7.13 (3.23-16.76)** | **0.017** |
| Double negative 4 B cells (cells/mm^3^) | 0.53 (0.24-1.11) | 0.43 (0-19-0-79) | 0.275 |
| IL-α (pg/mL) | 2.58 (0.55-15.34) | 2.81 (0.55-7.87) | 0.523 |
| IL-1β (pg/mL) | 0.80 (0.52-1.40) | 1.08 (0.68-1.69) | 0.192 |
| IL-1RA (pg/mL) | 25.60 (10.15-36-68) | 24.77 (11.85-58.77) | 0.389 |
| IL-2 (pg/mL) | 0.65 (0.42-1.02) | 0.60 (0.42-1.21) | 0.799 |
| IL-3 (pg/mL) | 0.13 (0.13-0.15) | 0.14 (0.13-0.16) | 0.118 |
| IL-4 (pg/mL) | 2.75 (2.74-218.40) | 2.75 (2.74-22.93) | 0.322 |
| IL-5 (pg/mL) | 1.20 (0.56-3.19) | 0.90 (0.65-1.26) | 0.348 |
| IL-6 (pg/mL) | 4.25 (1.29-20-13) | 5.62 (2.01-19.47) | 0.673 |
| IL-7 (pg/mL) | 10.08 (8.03-15.29) | 13.57 (8.87-18.61) | 0.109 |
| IL-8 (pg/mL) | 13.46 (8.44-20.57) | 13.19 (8.98-18.86) | 0.733 |
| **IL-10 (pg/mL)** | **5.83 (3.26-11.04)** | **10.79 (5.99-27.80)** | **0.007** |
| IL-12p70 (pg/mL) | 1.32 (1.32-1.69) | 1.32 (1.32-2.06) | 0.923 |
| IL-12p40 (pg/mL) | 1.49 (1.49-2.03) | 1.49 (1.49-1.67) | 0.695 |
| IL-13 (pg/mL) | 0.81 (0.75-5.77) | 0.81 (0.58-1.49) | 0.463 |
| **IL-15 (pg/mL)** | **4.11 (2.67-6.11)** | **6.07 (4.26-8.42)** | **0.006** |
| IL-17A (pg/mL) | 0.84 (0.11-2.13) | 0.76 (0.11-5.12) | 0.626 |
| TNF-α (pg/mL) | 22.33 (16.75-30.91) | 23.98 (17.24-32.58) | 0.450 |
| TNF-β (pg/mL) | 0.72 (0.52-18.84) | 0.64 (0.62-2.40) | 0.425 |
| G-CSF (pg/mL) | 23.16 (14.68-45.30) | 19.63 (14.68-56.37) | 0.969 |
| GM-CSF (pg/mL) | 2.66 (2.66-2.66) | 2.66 (2.66-4.36) | 0.294 |
| VEGF (pg/mL) | 76.17 (11.24-134.00) | 84.93 (41.55-202.04) | 0.224 |
| EGF (pg/mL) | 95.56 (56.89-143.25) | 78.69 (51.37-129.09) | 0.633 |
| IFN-α2 (pg/mL) | 13.79 (13.79-13.79) | 13.79 (13.79-13.79) | 0.564 |
| IFN-γ (pg/mL) | 4.49 (2.40-9.21) | 4.65 (2.11-18.53) | 0.346 |
| MCP-1/CCL2 (pg/mL) | 421.10 (257.60-592.20) | 425.50 (309.60-620.40) | 0.510 |
| MIP-1α /CCL3 (pg/mL) | 1.03 (1.03-2.24) | 1.03 (1.03-2.24) | 0.786 |
| MIP-1β /CCL4 (pg/mL) | 34.75 (25.43-45.39) | 39.57 (28.94-51.93) | 0.347 |
| **IP-10/CXCL10 (pg/mL)** | **663.90 (352.60-1049.20)** | **1039.00 (500.60-2528.60)** | **0.009** |
| Eotaxin/CCL11 (pg/mL) | 106.13 (76.03-145.34) | 108.61 (74.97-146.72) | 0.831 |
| NETs (AU) | 1.25 (0.93-1.61) | 1.29 (1.12-1.58) | 0.663 |
| Titer of AC antibodies (AU) | 80.00 (40.00-160.00) | 160.00 (80.00-320.00) | 0.663 |

EGF: epidermal growth factor; G-CSF: granulocyte colony-stimulating factor; GM-CSF: granulocyte-macrophage colony-stimulating factor; IFN: interferon; IL: interleukin; IP: IFN-γ-induced protein; IQR: interquartile range; MCP: monocyte chemoattractant protein; MFI: mean fluorescence intensity; MIP: macrophage inflammatory protein; TNF: tumor necrosis factor; VEGF: vascular endothelial growth factor.

**Supplementary Table 2.** Evaluation of the serum levels of cytokines, chemokines, NETs and AC antibodies 3 months after recruitment according to the development of a sustained humoral immune response. The serum levels of the cytokines, chemokines, NETs and the titers of AC antibodies are expressed as medians and interquartile ranges and were compared with the Wilcoxon test.

| **Variable** | **No sustained humoral immune response**  **Median (IQR)** | **Sustained humoral immune response**  **Median (IQR)** | **P-value** |
| --- | --- | --- | --- |
| IL-1α (pg/mL) | 1.91 (1.91-15.32) | 1.91 (1.91-2.20) | 0.141 |
| IL-1β (pg/mL) | 1.12 (0.94-1.64) | 1.28 (1.00-1.70) | 0.350 |
| IL-1RA (pg/mL) | 18.39 (11.02-27.59) | 16.95 (11.21-27.09) | 0.897 |
| **IL-2 (pg/mL)** | **0.91 (0.67-1.30)** | **1.11 (0.84-1.48)** | **0.048** |
| IL-3 (pg/mL) | 0.29 (0.27-0.31) | 0.31 (0.27-0.64) | 0.050 |
| IL-4 (pg/mL) | 28.84 (4.00-134.12) | 8.11 (4.00-40.52) | 0.057 |
| IL-5 (pg/mL) | 1.32 (0.95-2.43) | 1.21 (0.95-1.63) | 0.630 |
| IL-6 (pg/mL) | 2.16 (1.12-5.43) | 1.58 (2.26-3.01) | 0.376 |
| IL-7 (pg/mL) | 8.37 (5.68-11.88) | 7.22 (4.46-12.02) | 0.343 |
| IL-8 (pg/mL) | 6.65 (4.45-12.35) | 5.58 (4.08-8.79) | 0.397 |
| IL-10 (pg/mL) | 2.90 (1.82-4.32) | 3.16 (2.23-4.48) | 0.521 |
| IL-12p70 (pg/mL) | 1.65 (1.03-3.01) | 1.90 (1.23-2.69) | 0.532 |
| IL-12p40 (pg/mL) | 0.27 (0.27-2.07) | 0.82 (0.27-6.94) | 0.187 |
| IL-13 (pg/mL) | 1.93 (0.48-9.75) | 1.15 (0.62-2.62) | 0.429 |
| IL-15 (pg/mL) | 1.74 (1.29-2.16) | 2.00 (1.52-2.82) | 0.182 |
| IL-17A (pg/mL) | 2.73 (1.63-3.62) | 2.54 (1.76-4.19) | 0.486 |
| TNF-α (pg/mL) | 12.01 (10.12-14.53) | 13.03 (9.73-16.57) | 0.495 |
| TNF-β (pg/mL) | 1.82 (0.96-22.80) | 1.75 (0.82-3.58) | 0.370 |
| G-CSF (pg/mL) | 11.73 (4.36-24.45) | 13.12 (4.80-21.80) | 0.823 |
| GM-CSF (pg/mL) | 1.01 (0.34-1.96) | 1.36 (0.80-2.85) | 0.099 |
| VEGF (pg/mL) | 56.10 (36.91-99.09) | 68.65 (44.55-95.62) | 0.479 |
| EGF (pg/mL) | 54.66 (27.39-102.04) | 59.63 (32.33-148.11) | 0.285 |
| IFN-α2 (pg/mL) | 4.22 (4.22-4.22) | 4.22 (4.22-4.22) | 0.380 |
| IFN-γ (pg/mL) | 5.48 (2.16-11.10) | 5.19 (2.58-10.04) | 0.635 |
| MCP-1/CCL2 (pg/mL) | 327.60 (243.70-491.60) | 395.20 (303.40-492.80) | 0.211 |
| MIP-1α /CCL3 (pg/mL) | 2.42 (2.42-2.42) | 2.42 (2.42-2.42) | 0.314 |
| MIP-1β /CCL4 (pg/mL) | 24.95 (16.02-30.88) | 25.68 (16.65-35.36) | 0.471 |
| IP-10/CXCL10 (pg/mL) | 313.12 (224.28-429.39) | 320.30 (257.48-447.62) | 0.547 |
| Eotaxin/CCL11 (pg/mL) | 82.49 (51.56-133.12) | 88.00 (73.49-119.21) | 0.393 |
| NETs (AU) | 0.94 (0.86-1.07) | 0.96 (0.87-1.33) | 0.269 |
| Titer of AC antibodies (AU) | 16.00 (80.00-181.10) | 157.20 (80.00-160.00) | 0.831 |

AC: anti-cellular; EGF: epidermal growth factor; G-CSF: granulocyte colony-stimulating factor; GM-CSF: granulocyte-macrophage colony-stimulating factor; IFN: interferon; IL: interleukin; IP: IFN-γ-induced protein; IQR: interquartile range; MCP: monocyte chemoattractant protein; MIP: macrophage inflammatory protein; NETs: neutrophil extracellular traps; TNF: tumor necrosis factor; VEGF: vascular endothelial growth factor.

**Supplementary Table 3**. Comparison of the serum levels of cytokines, chemokines and AC antibodies in patients with COVID-19, 6 months after recruitment according to the diagnosis of a sustained humoral immune response. The serum levels of the cytokines and chemokines are expressed as medians and interquartile ranges and were compared with the Wilcoxon test.

| **Variable** | **No sustained humoral immune response**  **Median (IQR)** | **Sustained humoral immune response**  **Median (IQR)** | **P-value** |
| --- | --- | --- | --- |
| IL-1α (pg/mL) | 1.36 (1.36-24.09) | 1.36 (1.36-6.18) | 0.330 |
| IL-1β (pg/mL) | 1.15 (0.71-1.88) | 1.19 (0.75-2.11) | 0.560 |
| IL-1RA (pg/mL) | 22.58 (15.72-39.41) | 22.97 (13.67-44.36) | 0.986 |
| IL-2 (pg/mL) | 0.49 (0.10-0.94) | 0.53 (0.13-1.14) | 0.626 |
| **IL-3 (pg/mL)** | **0.04 (0.03-0.05)** | **0.05 (0.04-0.42)** | **0.015** |
| IL-4 (pg/mL) | 1.40 (1.40-253.80) | 4.72 (1.40-59.60) | 0.487 |
| IL-5 (pg/mL) | 5.60 (5.60-5.60) | 5.60 (5.60-5.60) | 0.343 |
| IL-6 (pg/mL) | 1.00 (1.00-12.56) | 1.00 (1.00-4.55) | 0.168 |
| IL-7 (pg/mL) | 12.67 (4.66-17.58) | 10.65 (6.47-16.71) | 0.986 |
| IL-8 (pg/mL) | 8.34 (5.96-20.67) | 9.62 (5.23-14.16) | 0.772 |
| IL-10 (pg/mL) | 0.91 (0.23-3.58) | 1.89 (0.25-3.85) | 0.256 |
| IL-12p70 (pg/mL) | 1.30 (1.30-3.99) | 2.33 (1.30-3.65) | 0.488 |
| **IL-12p40 (pg/mL)** | **0.31 (0.31-4.36)** | **4.36 (0.31-13.80)** | **0.041** |
| IL-13 (pg/mL) | 1.49 (1.49-16.53) | 1.49 (1.49-1.51) | 0.073 |
| IL-15 (pg/mL) | 1.55 (1.55-1.55) | 1.55 (1.55-2.70) | 0.220 |
| IL-17A (pg/mL) | 0.52 (0.52-3.25) | 0.52 (0.52-5.02) | 0.235 |
| TNF-α (pg/mL) | 14.82 (13.32-19.61) | 18.11 (13.17-20.11) | 0.196 |
| TNF-β (pg/mL) | 2.24 (2.24-62.85) | 2.24 (2.24-5.93) | 0.195 |
| G-CSF (pg/mL) | 22.32 (14.12-36.22) | 22.32 (13.22-35.77) | 0.839 |
| GM-CSF (pg/mL) | 1.61 (1.61-2.42) | 1.61 (1.61-2.78) | 0.959 |
| VEGF (pg/mL) | 97.17 (68.59-125.42) | 102.64 (68.93-143.00) | 0.397 |
| EGF (pg/mL) | 85.63 (30.63-162.63) | 101.93 (42.08-207.53) | 0.288 |
| IFN-α2 (pg/mL) | 4.60 (4.60-4.60) | 4.60 (4.60-4.60) | 0.731 |
| IFN-γ (pg/mL) | 7.59 (4.06-10.93) | 7.94 (5.57-13.54) | 0.395 |
| **MCP-1/CCL2 (pg/mL)** | **364.20 (270.90-501.40)** | **442.60 (378.50-567.50)** | **0.040** |
| MIP-1α /CCL3 (pg/mL) | 1.85 (1.85-1.85) | 1.85 (1.85-1.85) | 0.275 |
| **MIP-1β /CCL4 (pg/mL)** | **29.19 (22.16-35.91)** | **33.08 (26.12-44.79)** | **0.024** |
| IP-10/CXCL10 (pg/mL) | 302.90 (205.50-426.10) | 328.13 (257.65-412.50) | 0.374 |
| Eotaxin/CCL11 (pg/mL) | 106.58 (78.25-157.56) | 119.81 (95.06-155.55) | 0.297 |
| Titer of AC antibodies (AU) | 80.00 (80.00-283.00) | 160 (80.00-320.00) | 0.298 |

EGF: epidermal growth factor; G-CSF: granulocyte colony-stimulating factor; GM-CSF: granulocyte-macrophage colony-stimulating factor; IFN: interferon; IL: interleukin; IP: IFN-γ-induced protein; IQR: interquartile range; MCP: monocyte chemoattractant protein; MIP: macrophage inflammatory protein; TNF: tumor necrosis factor; VEGF: vascular endothelial growth factor.

**Supplementary** **Table 4.** Univariate logistic regression analysis of the statistically significant features associated to the development of a sustained humoral immune response in patients with COVID-19

| **Variable** | **OR (95% confidence interval)** | **P-value** |
| --- | --- | --- |
| **Baseline** | | |
| Absolute number of monocytes (cells/mm^3^) | 0.995 (0.989–0.999) | 0.031742 |
| Arterial lactate (mmol/L) | 0.002 (0.001×10^−4^–0.076×10^−4^) | 0.028786 |
| Alkaline phosphatase (U/L) | 0.974 (0.907–0.990) | 0.002818 |
| TNF-β (pg/mL) | 0.896 (0.767–0.981) | 0.010369 |
| IL-8 (pg/mL) | 0.876 (0.743–0.974) | 0.005903 |
| IL-5 (pg/mL) | 0.689 (0.392–0.934) | 0.042105 |
| IL-4 (pg/mL) | 0.988 (0.954–0.997) | 0.028248 |
| IL-1α (pg/mL) | 0.915 (0.790–0.986) | 0.024247 |
| IL-13 (pg/mL) | 0.704 (0.426–0.950) | 0.012660 |
| Pre-switched memory B cells (cells/mm^3^) | 0.164 (0.0163–0.786) | 0.025966 |
| Double-negative B cells (cells/mm^3^) | 0.794 (0.577–0.981) | 0.037849 |
| **Three months after recruitment** | | |
| IL-7 (pg/mL) | 1.284 (1.016–1.714) | 0.035755 |
| IL-6 (pg/mL) | 1.286 (1.030–1.885) | 0.017968 |
| IL-4 (pg/mL) | 1.011 (1.001–1.029) | 0.009976 |
| IL-1α (pg/mL) | 1.071 (1.005–1.169) | 0.018150 |
| IL-13 (pg/mL) | 1.142 (1.0132–1.376) | 0.015937 |
| Anti-SARS-CoV-2 IgG (AU) | 2.991 (1.095–11.373) | <0.0001 |
| Neutrophil extracellular traps (AU) | 0.002 (0.001×10^−3^–0.903) | 0.046123 |
| **Six months after recruitment** | | |
| TNF-β (pg/mL) | 0.974 (0.936–0.994) | 0.034403 |
| IL-6 (pg/mL) | 0.928 (0.842–0.992) | 0.028886 |
| IL-4 (pg/mL) | 0.996 (0.990–0.999) | 0.023959 |
| IL-1α (pg/mL) | 0.971 (0.932–0.995) | 0.029596 |

AU: arbitrary units; COVID-19: coronavirus disease 2019; IgG: immunoglobin G; IL: interleukin; ODI: optical density index; OR: odds ratio; SARS-CoV-2: severe acute respiratory syndrome coronavirus 2; TNF-β: tumor necrosis factor-β.

**Supplementary table 5.** Repeated-measures analysis of cytokines by disease severity and development of a sustained humoral immune response. The results from the univariate generalized linear mixed model with binomial error are depicted. Post-hoc comparisons were performed for significant variables at 6 months after recruitment

| **Variable** | **Critical disease without SHIR**  **Median (IQR)** | **Critical disease with SHIR**  **Median (IQR)** | **Non-critical disease without SHIR**  **Median (IQR)** | **Non-critical disease with SHIR**  **Median (IQR)** | **P-value** |
| --- | --- | --- | --- | --- | --- |
| TNF-β (pg/mL) | 98.75 (71.16–126.34) | 7.16 (4.61–18.94) | 18.80 (8.01–29.60) | 14.29 (4.37–24.21) | <0.0001 |
| IL-5 (pg/mL) | 8.78 (5.55–12.02) | 2.70 (0.04–5.44) | 3.62 (0.89–6.35) | 3.38 (0.67–6.09) | <0.0001 |
| IL-3 (pg/mL) | 0.26 (0.08–0.44) | 0.37 (0.20–0.33) | 0.17 (0.01–0.33) | 0.17 (0.01–0.33) | <0.0001 |
| IL-1 (pg/mL) | 95.53 (74.37–116.69) | 7.17 (0.30–14.65) | 15.72 (9.25–22.20) | 12.16 (6.63–17.68) | <0.0001 |
| IL-13 (pg/mL) | 37.94 (25.79–50.08) | 4.23 (0.95–9.42) | 9.14 (4.38–13.90) | 6.28 (1.91–10.65) | <0.0001 |

IL: interleukin; SHIR: sustained humoral immune response; TNF-β: tumour necrosis factor-β.

**Supplementary Figure 1.** Gating strategy for the evaluation of T helper, cytotoxic, naïve, exhausted, anergic, regulatory, senescent cell subsets


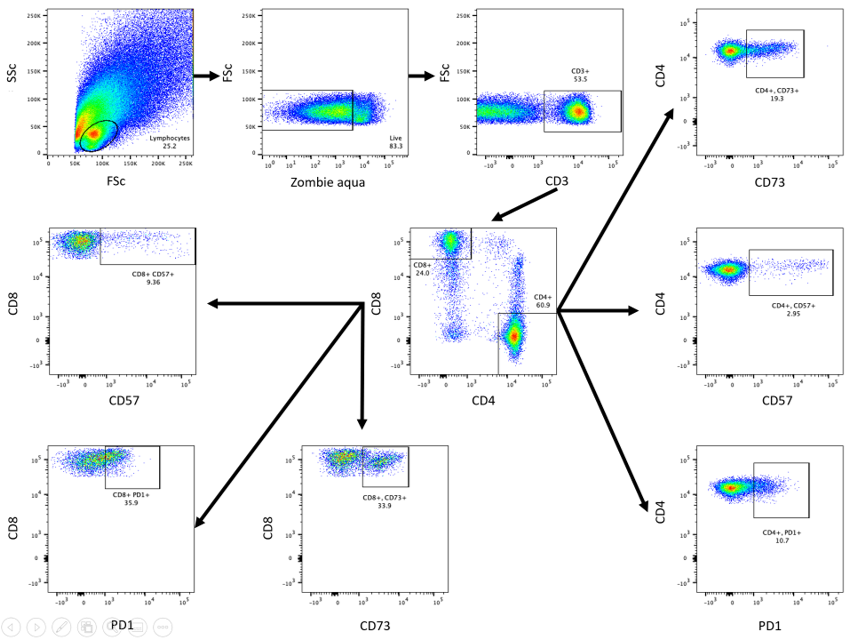


**Supplementary Figure 2.** Gating strategy for the evaluation of T memory cell subsets


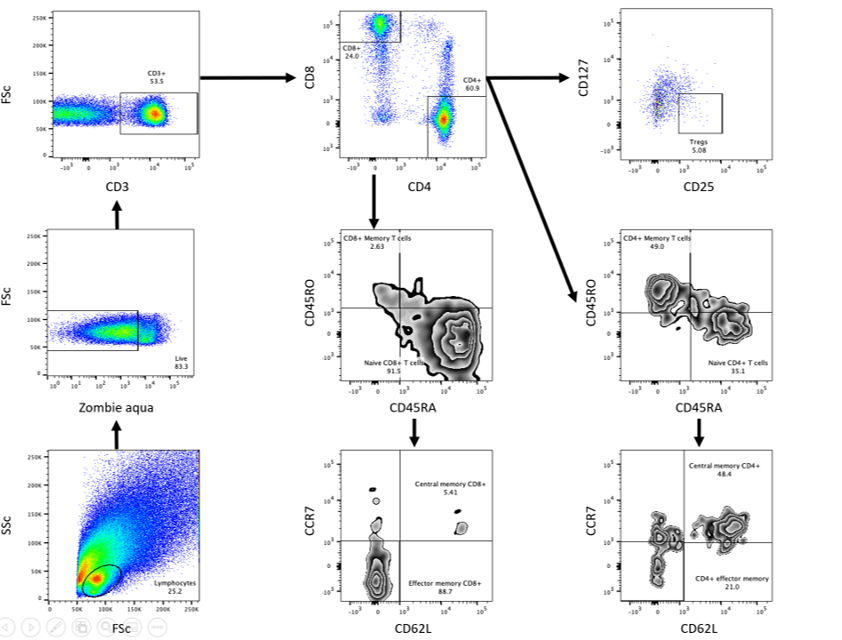


**Supplementary Figure 3.** Gating strategy for the evaluation of CD4^+^ Th1, Th2, Th17 and CD8^+^ Th1, Th2, Th17 cell subsets


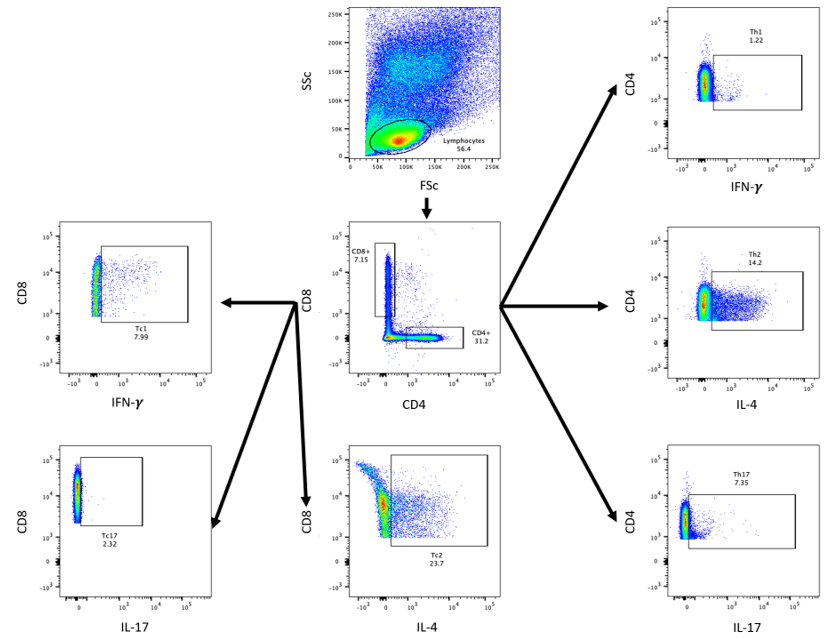


**Supplementary Figure 4.** Gating strategy for the evaluation of B cell subsets


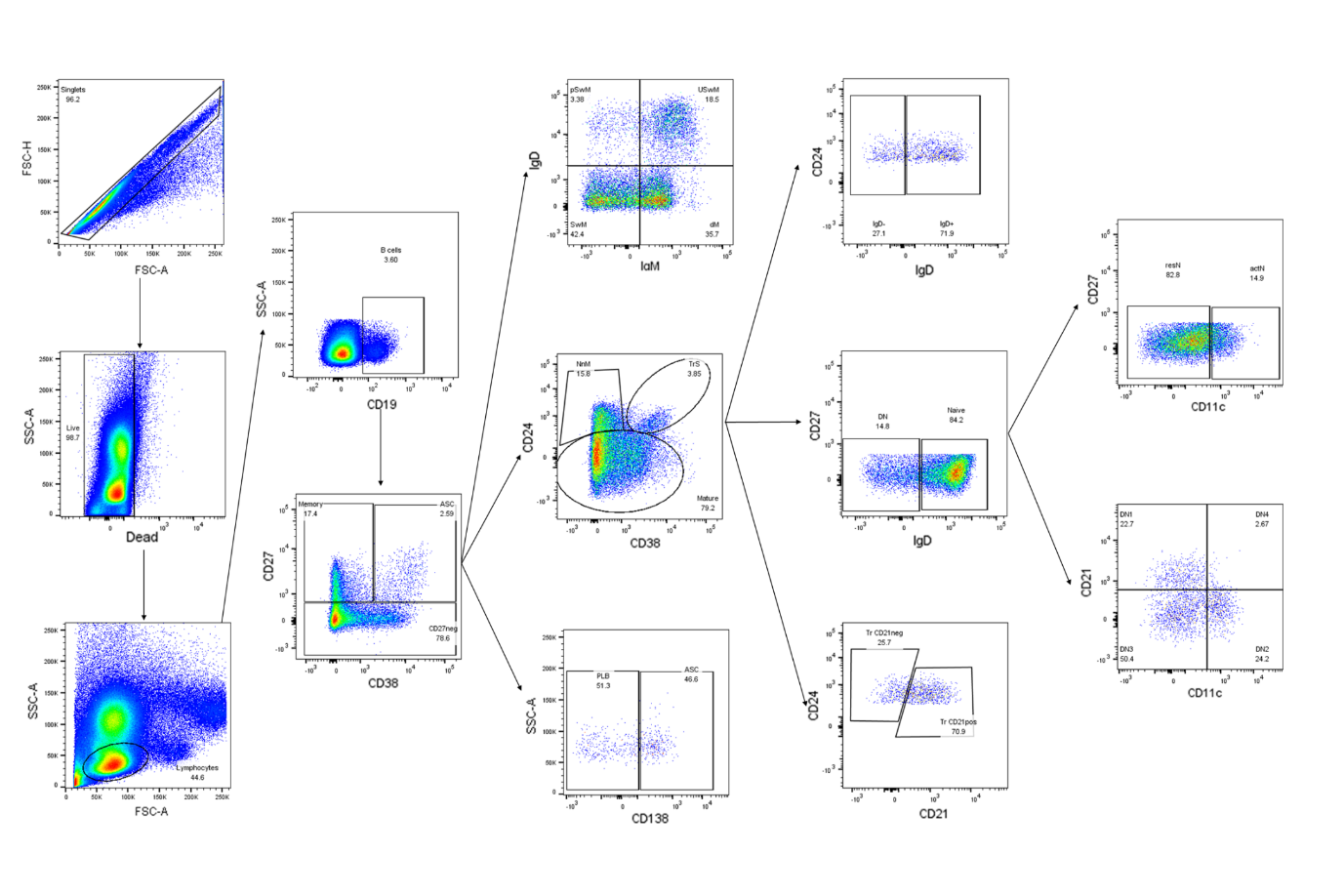


**Supplementary table 5.** Repeated-measures analysis (A-E) of the differential serum levels of cytokines 6 months after recruitment considering the disease severity and the development of a sustained humoral immune response


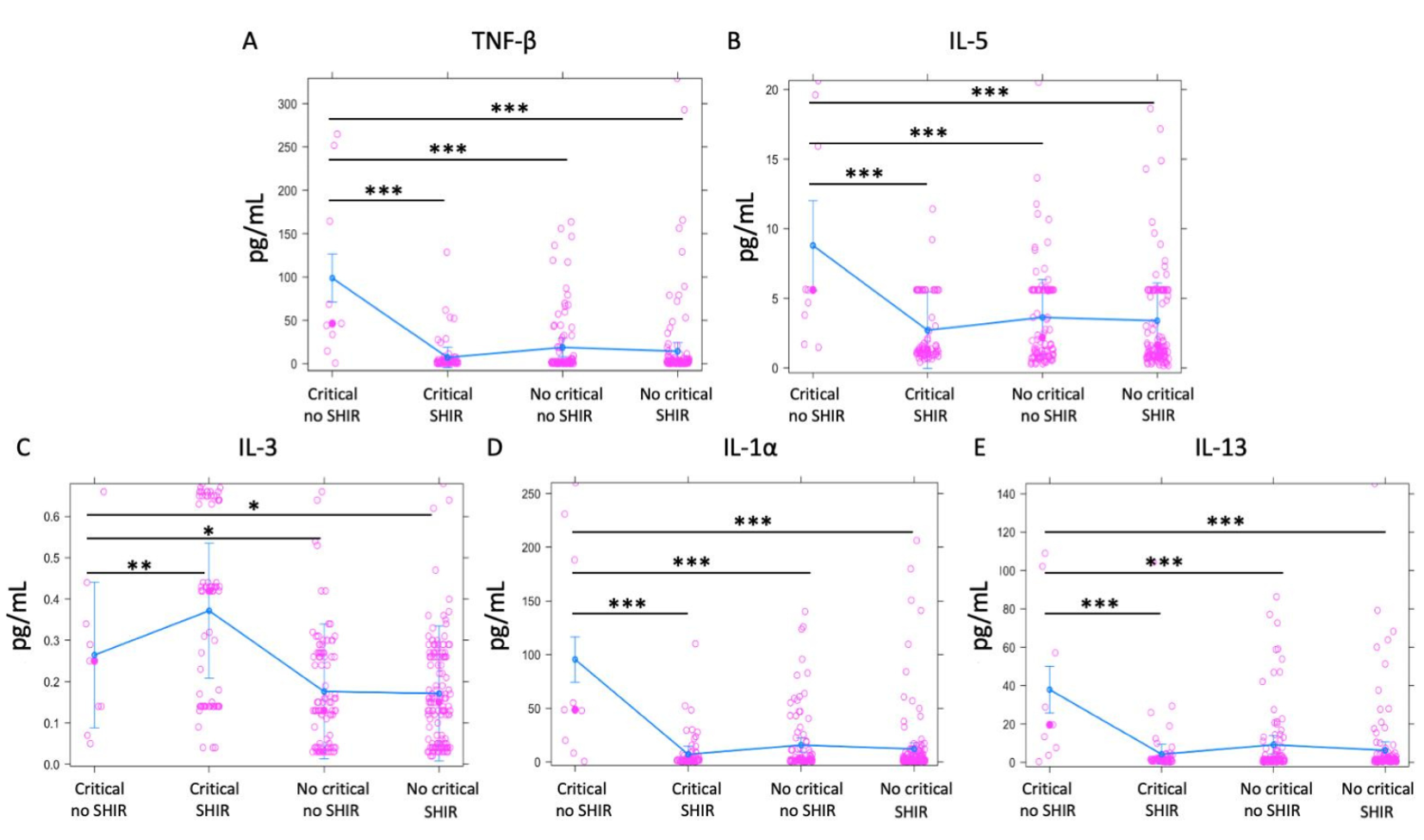

Supplement: Supplementary Figure 1 — Gating strategy for the evaluation of T helper, cytotoxic, naïve, exhausted, anergic, regulatory, senescent cell subsets. [file DataSheet_1.docx]
